# GADF-enhanced global–local feature network for wearable sensor-based cow behavior recognition

**DOI:** 10.3389/fvets.2026.1872214

**Published:** 2026-07-28

**Authors:** Chunjie Wang

**Affiliations:** School of Computer and Software Engineering, Chengdu Jincheng College, Chengdu, China

**Keywords:** cow behavior recognition, deep learning, IMU, inertial measurement unit, wearable sensors

## Abstract

**Introduction:**

Continuous and reliable monitoring of dairy cow behaviors is a key component of data-driven herd management systems. Although wearable inertial sensors provide a practical alternative to camera-based monitoring, effectively representing complex motion patterns from raw inertial signals remains a challenge.

**Methods:**

This work presents an Inertial Measurement Unit (IMU)-based cow behavior recognition approach that emphasizes multi-scale motion representation using nose-mounted sensing. Acceleration data acquired from a nose-attached inertial measurement unit are utilized to capture characteristic head movement patterns associated with feeding, rumination, and locomotion activities. Instead of directly processing one-dimensional time-series signals, the inertial sequences are restructured into two-dimensional pseudo-color matrices through Gramian Angular Difference Field (GADF) transformation, allowing temporal correlations to be explicitly preserved. To exploit motion information at different levels of granularity, a multi-scale feature modeling network is developed, wherein complementary motion cues are extracted and integrated via a cross-attention-driven interaction mechanism.

**Results:**

Experimental results demonstrate that the proposed framework achieves competitive recognition performance, with an overall accuracy of 90.12%, and maintains robustness across different behavioral categories.

**Discussion:**

These findings confirm that transforming inertial signals into GADF-based pseudo-color images, coupled with multi-scale feature modeling, offers an effective and robust solution for automated cow behavior recognition.

## 1 Introduction

The global dairy industry is rapidly transitioning toward large-scale and intelligent production systems driven by increasing demand, stricter quality requirements, and rising costs (1). As the fundamental unit of production, dairy cow health and behavior directly affect milk yield, quality, and overall farm profitability. Key behaviors such as feeding, rumination, and moving serve as important indicators of physiological status (2). Abnormal changes in these behaviors are often associated with diseases or metabolic disorders (3). Therefore, accurate behavior recognition is essential for early disease detection, efficient herd management, and improved animal welfare, forming a critical component of precision livestock farming. Traditional behavior monitoring relies on manual observation, which is labor-intensive, subjective, and lacks continuity, making it unsuitable for large-scale and long-term monitoring (4). With the advancement of sensor technologies and artificial intelligence, automated monitoring systems have emerged as a promising solution. Existing methods can be broadly categorized into vision-based approaches and wearable sensor–based approaches (5).

### 1.1 Vision-based cow behavior recognition methods

With the rapid development of computer vision and deep learning, vision-based cow behavior recognition has become an important research direction in precision dairy farming (6). These methods utilize cameras to capture images or video sequences of cow activities and apply learning-based models to extract behavioral features for classification. Vision-based systems offer advantages such as non-contact monitoring and intuitive visual interpretation (7), enabling continuous observation of individual or group behaviors in farm environments. Early approaches relied on traditional computer vision techniques, including image segmentation, background modeling, and motion detection, which depend heavily on handcrafted features and are sensitive to environmental variations such as illumination changes and occlusions. Recent advances in deep learning have significantly improved performance, with models such as convolutional neural networks (CNNs), long short-term memory networks (LSTM), and spatiotemporal graph convolutional networks (ST-GCN) enabling automatic extraction of discriminative spatiotemporal features (8). In addition, related progress in object detection and visual recognition—such as you only look once (YOLO)-based detection frameworks, attention-enhanced networks, transformer-based architectures, and domain adaptation approaches—has further improved detection robustness and real-time performance in complex visual scenes (9–11, 35–39). These methods have demonstrated strong performance under controlled experimental conditions.

However, vision-based approaches still face critical challenges in real-world deployment. Their performance is often affected by complex environmental factors, including lighting variations, occlusions, and high-density scenes. In addition, high installation and maintenance costs, as well as the large computational and storage requirements of video data, limit their scalability and real-time applicability in commercial farms. Therefore, enhancing robustness, cost-efficiency, and deployment feasibility remains a key challenge for vision-based cow behavior recognition systems.

### 1.2 Wearable sensor-based cow behavior recognition methods

To address the limitations of vision-based monitoring, wearable sensor–based approaches have emerged as a promising alternative for cow behavior recognition (12). These systems collect behavior-related physical signals, such as acceleration, pressure, temperature, and acoustic data, through sensors attached to different body locations (e.g., collars, ear tags, leg bands, or nose rings), and perform behavior classification via signal processing and data-driven models. Compared with vision-based methods, wearable sensing systems offer stronger environmental robustness, lower deployment cost, and better real-time performance, making them well-suited for large-scale farm applications. Among various sensing modalities, tri-axial accelerometers are the most widely adopted due to their low power consumption, high sensitivity, and effectiveness in capturing motion dynamics (13). In addition, multimodal sensing strategies that integrate complementary signals (e.g., acoustic or physiological data) have been explored to further enhance behavioral representation.

Regarding sensor placement, existing studies have primarily focused on the neck, legs, and ears of cows. Among these, neck-mounted wearable devices represent the most mature solution and can integrate multiple sensors to enable multidimensional data acquisition, offering broad applicability (14). However, neck movements are only weakly correlated with core physiological behaviors such as feeding and rumination, resulting in low recognition accuracy. Leg-mounted devices primarily monitor step count and movement intensity and are commonly used for detecting estrus-related activity as well as behaviors such as walking and lying (15). Ear-mounted devices are characterized by their small size, ease of attachment, and minimal interference with daily activities, and are often used for individual identification and basic behavior monitoring (16). In terms of behavior recognition algorithms, early studies predominantly adopted traditional machine learning methods, such as support vector machines (SVM), decision trees, and k-nearest neighbors (KNN) (17, 18). In recent years, with the rapid development of deep learning—particularly CNNs, LSTM, and transformer for time-series data analysis—the automatic feature extraction and behavior recognition capabilities of wearable sensor signals have been significantly enhanced (19, 20). For example, Kumar et al. (21) proposes an IoT-enabled convolutional transformer that integrates Bi-GRU-based temporal modeling, spatial attention, and a temporal adaptive transformer with continual learning for robust dairy farming monitoring and real-time behavior detection. Zhao et al. (22) compares Transformer, CNN-Transformer, and LSTM-Transformer models for dairy cow calving time prediction using tail acceleration data, to address class imbalance, and reports competitive performance across models with accuracies around 0.93–0.94.

Despite the substantial progress achieved by wearable sensor-based monitoring methods, two key challenges remain. First, existing sensor placements (e.g., neck, legs, or ears) are often insufficient to capture high-fidelity signals strongly correlated with core physiological behaviors such as feeding and rumination, leading to suboptimal recognition performance. Second, current data processing and feature learning methods remain limited in their ability to fully exploit the multi-scale and discriminative information embedded in raw behavioral signals, as many studies still rely on traditional machine learning or single-dimensional representations.

### 1.3 This work

Despite recent progress in IMU-based behavior recognition, two key limitations remain: (1) current sensor placements often fail to capture highly discriminative signals strongly correlated with feeding and rumination behaviors; and (2) most existing methods rely on 1D time-series modeling and struggle to fully exploit multi-scale temporal–spatial representations. To address these challenges, this study proposes an integrated cow behavior recognition framework based on nose-mounted IMU signals. The framework captures motion patterns closely related to feeding, rumination, and moving, and transforms raw signals into discriminative time-series images for multi-scale feature learning. The main contributions are summarized as follows:

- Tri-axial acceleration data from a nose-mounted IMU are utilized to monitor cow behaviors, enabling non-intrusive, real-time observation of feeding, rumination, and moving. By leveraging the strong coupling between nasal motion and oral activities, this data provides enhanced observability of core physiological behaviors compared with conventional wearable placements.
- A time-series-to-image encoding strategy based on GADF is introduced to transform preprocessed acceleration sequences into two-dimensional pseudo-color images. This representation effectively preserves temporal dependencies and dynamic variation patterns within behavioral signals, allowing the cow behavior recognition task to be reformulated as an image classification problem and facilitating the use of advanced convolutional neural networks.
- A dual-stream network architecture is developed to simultaneously extract and enhance global and local behavior-related features from the encoded images. Specifically, the proposed network consists of a global feature extraction branch equipped with an attention-based feature enhancement module, and a local feature extraction branch guided by an attention activation mechanism and region selection strategy. In addition, a Multi-Head Cross-Attention Coupling module is designed to model the interaction between global and local representations, enabling adaptive feature fusion and comprehensive multi-scale representation learning.

## 2 Materials and methods

### 2.1 Wearable cow behavior recognition system

**Figure 1:**
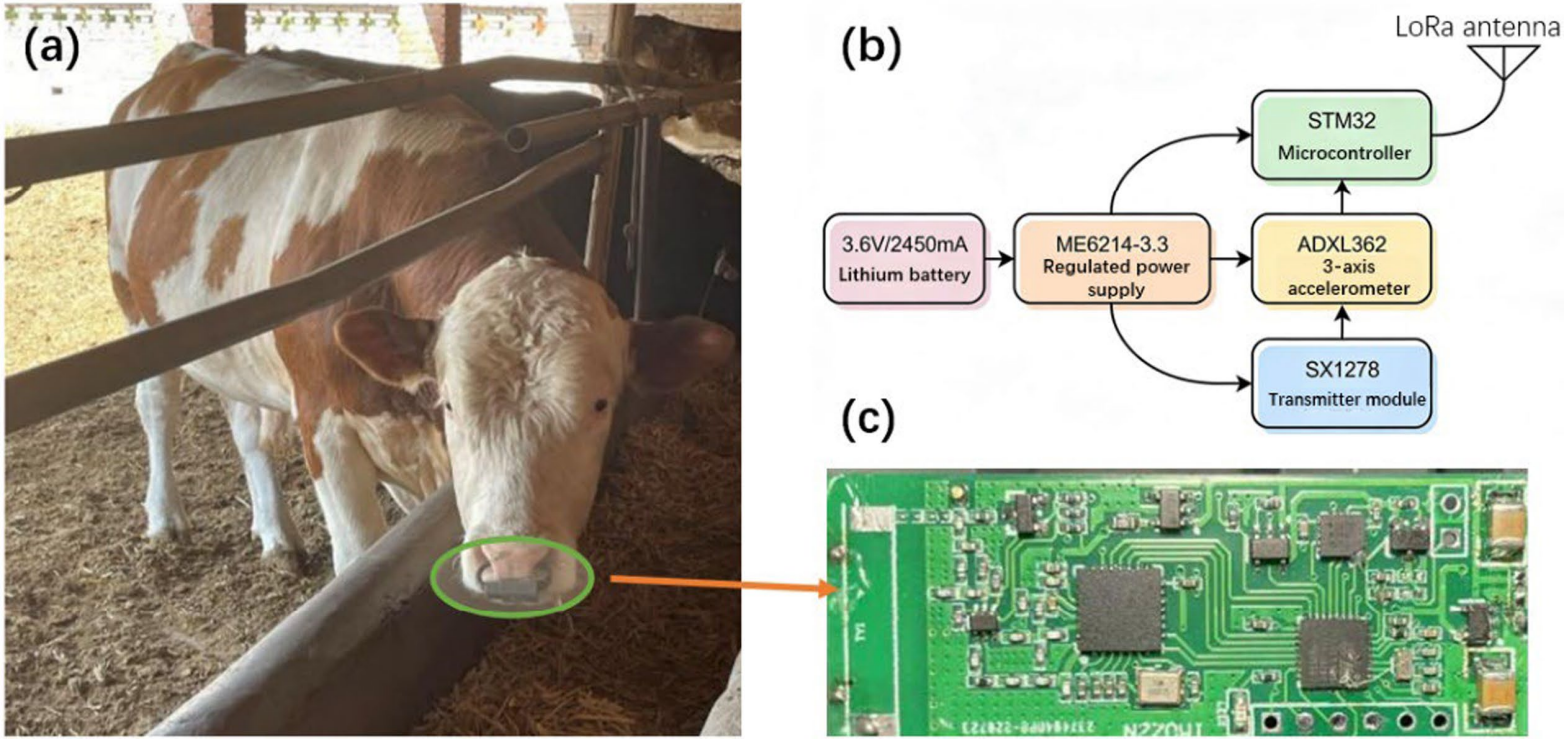
**(a)** Wearing method of the wearable sensor, **(b)** structural components of the wearable sensor, **(c)** sensor circuit board.

As illustrated in Figure 1 the wearable cow behavior recognition system based on sensors consists of three main components: a wearable data acquisition terminal mounted on the cow’s nose, a LoRa wireless communication gateway, and a remote server. The system is designed to achieve non-intrusive, real-time monitoring and recognition of three key behaviors: feeding, rumination, and moving.

The data acquisition terminal mainly integrates a microcontroller, a LoRa communication module, an ADXL362 accelerometer, and a power supply system (23). At its core, an ultra-low-power STM32L051K8 microcontroller handles overall system scheduling and data processing. The behavior sensing module employs the ADXL362 three-axis accelerometer from Analog Devices, which features ultra-low power consumption (typical ≤2 μA at 100 Hz sampling rate), high resolution (1 mg/LSB at ±2 g range), and compact size (3 mm × 3.25 mm × 1.06 mm), making it well-suited for long-term wear. The sensor collects raw 3D acceleration data from the cow’s nose at a frequency of 10 Hz, allowing sensitive detection of motion patterns related to chewing, rumination, and general body movement. Data are transmitted to the microcontroller via the SPI interface and temporarily stored in the sensor’s built-in FIFO buffer.

The communication module uses a LoRa unit based on the SX1278 chip to wirelessly transmit the processed data. The power supply module consists of a 3.6 V / 2,450 mAh disposable lithium battery, paired with an ME6214 low-dropout regulator to provide a stable 3.3 V output. This configuration ensures long-term operation (continuous use for 5–6 h during experiments) while meeting the requirements for lightweight and compact device design.

The system adopts a low-power intermittent operation mode to optimize energy consumption. The microcontroller wakes up every 2 s and, during this period, reads the 20 sets of (X, Y, Z) acceleration data accumulated over the past 2 s from the ADXL362 FIFO buffer. These data are then packaged into a specific frame format, including a frame header, device ID, acceleration data payload, and frame tail, and transmitted via the LoRa module to the LoRa gateway deployed in the farm area.

The gateway is built around an STM32F103C8T6 microcontroller, a LoRa receiving module, and a 4G communication module (JL-10 T Plus). Upon receiving data from multiple nose-ring terminals, the gateway performs checksum verification and format conversion before uploading the processed data to a cloud server or a local monitoring workstation via the 4G network, completing a closed data loop from the sensing nodes to the cloud platform.

### 2.2 Data collection protocol

#### 2.2.1 Definition of cow behaviors

Animal welfare can be widely threatened by factors such as feed-induced gastrointestinal discomfort or sudden temperature drops, which may lead to disease. Changes in animal behavior are closely linked to production, health, and environmental conditions. Cow behaviors include basic actions (standing, walking, lying), rumination, lameness, etc. Based on on-site observations, the behaviors of cows in this study are categorized into three types: feeding, rumination, and moving.

Feeding: Defined as the cow standing still with legs straight and bending its neck to eat. Normal daily feeding duration and frequency fall within a specific range. Excessive feeding within a day may indicate estrus, while a sharp decrease may suggest illness.

Rumination: Defined as the cow standing still or lying down while repeatedly chewing cud. Normal rumination duration and frequency also have typical ranges. A sudden increase may indicate nutritional deficiency, whereas a sharp decrease may suggest estrus.

Moving: Defined as movement involving alternating leg steps, rapid walking, head shaking, or other behaviors not classified as feeding or rumination, including standing still to drink water. Cows naturally exhibit relatively low activity; a daily increase in moving may indicate estrus. During pregnancy, increased moving is harmful. Therefore, monitoring and controlling moving frequency is important for improving calving rates and supporting milk production.

#### 2.2.2 Data collection

**Figure 2:**
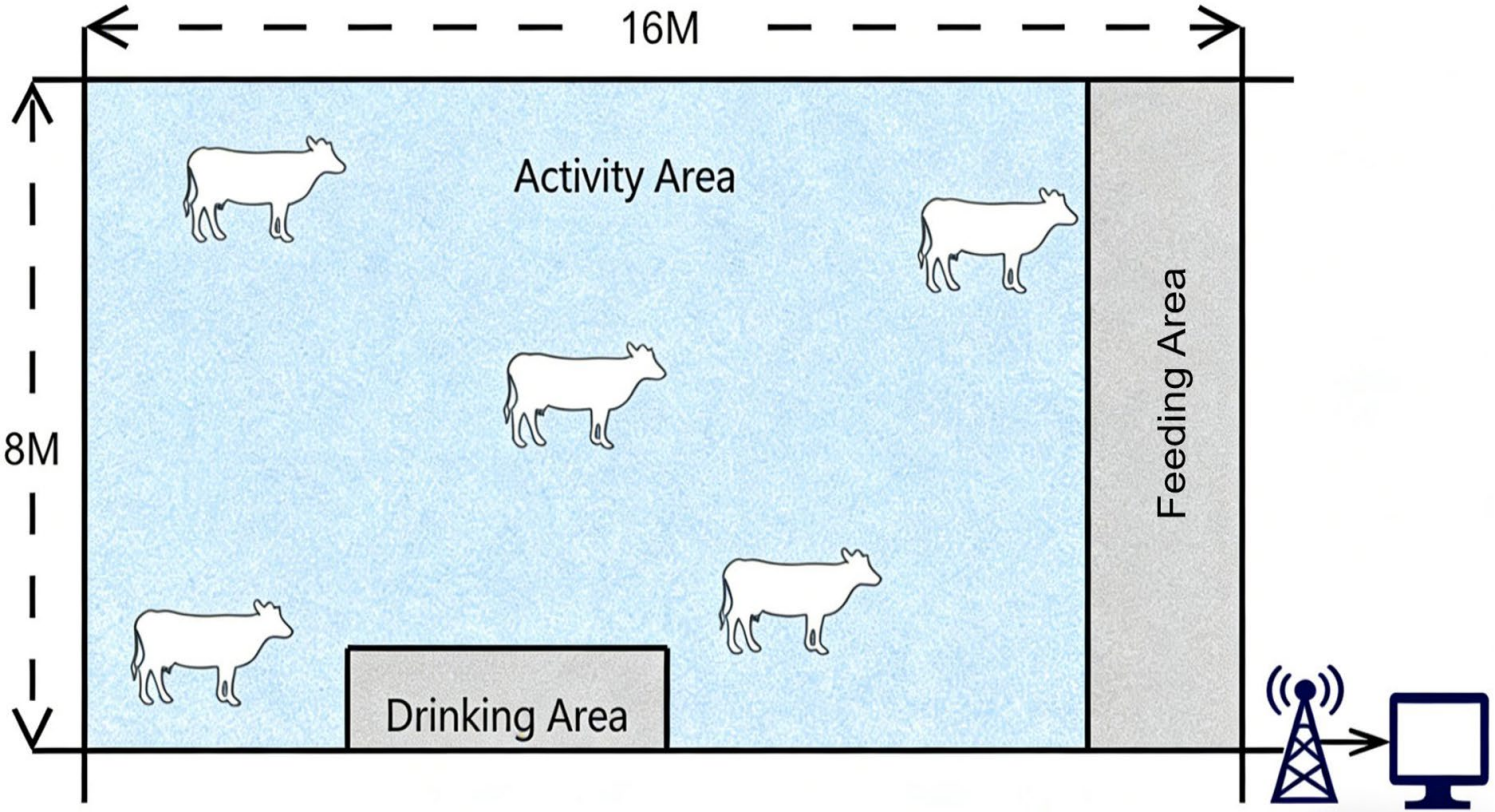
Map of the experimental dairy farm.

The data used in this study were obtained from an 11-day on-site data collection experiment conducted in June 2023 at a dairy farm in Hohhot, Inner Mongolia (see Figure 2 for the experimental area). All experimental protocols were approved by the Animal Ethics Committee of Inner Mongolia University (Approval No. 20230089). This study was carried out in accordance with relevant guidelines and regulations. Seven clinically healthy adult Holstein dairy cows were selected as experimental subjects from a commercial dairy farm in Hohhot, Inner Mongolia, China. All cows were pre-screened by a professional veterinarian to ensure the absence of locomotor disorders, metabolic diseases, mastitis, or abnormal feeding behavior. Only animals with stable milk production records and no illness or medication history within the past 2 weeks were included to ensure experimental consistency. During each experimental session, cows wore a lightweight, non-invasive nose-ring IMU device for approximately 5–6 h (8:00 a.m. to 1:00 p.m.). A 30-min adaptation period was provided prior to data collection to reduce potential stress. Animal welfare was monitored throughout the experiment by trained staff, focusing on signs of discomfort, abnormal posture, reduced activity, or feeding interruption. All animals were kept under standard farm conditions with free access to water and TMR feeding. The study was approved by the Animal Ethics Committee of Inner Mongolia University (No. 20230089) and complied with the 3R principles.

High-definition cameras (resolution: 2560 × 1920, frame rate: 25 fps) were deployed at multiple fixed positions around the cow activity area to continuously record video of the experimental cows wearing the nose-ring IMU devices. The camera views were adjusted to ensure full coverage of feeding, rumination, and movement areas without obstructing natural behavior. To ensure temporal alignment between sensor signals and video recordings, the timestamps of the IMU data acquisition system and the video recording system were manually synchronized at the beginning of each experimental session using a shared reference time point. This enabled accurate correspondence between sensor data segments and behavioral events observed in the video. Behavior labels were assigned based on synchronized video analysis, and three categories were defined: 0 (feeding), 1 (rumination), and 2 (moving). Two independent annotators with experience in animal behavior analysis were involved in the labeling process. In cases of disagreement, a consensus discussion was conducted to determine the final label. Inter-annotator reliability was quantified using Cohen’s kappa coefficient to ensure labeling consistency and reliability. The results show a high level of consistency between annotators (*κ* = 0.91), indicating that the labeling process is reliable and suitable for model training and evaluation. This level of agreement is considered reasonable given that occasional ambiguity exists in transitional behavioral segments (e.g., between feeding and rumination), where similar motion patterns may introduce minor subjective differences in annotation.

### 2.3 Signal preprocessing

*Data segmentation:* The raw acceleration data are continuous time series. To convert them into independent samples suitable for model input, a sliding window technique is applied. First, the synchronized and aligned video annotation information is mapped onto the continuous data stream to ensure that each data point is associated with a corresponding behavior label (feeding, rumination, moving). Then, a sliding window of 6.4 s is used to extract typical behavior segments. Each extracted segment contains the raw acceleration values along the X, Y, and Z axes, forming a 64 × 3 matrix.

*Data filtering:* Due to natural cow movements and potential device-related interference, the raw acceleration signals contain high-frequency noise. To extract low-frequency components that reflect true macro-movements of cows, a low-pass filter is applied to each axis of the acceleration sequence. Specifically, a second-order Butterworth low-pass filter with a cutoff frequency of 5 Hz is used. The transfer function of the filter in the S-domain is given by Equation 1:

$$H(s)=\frac{\omega_{c}^{2}}{s^{2}+\sqrt{2}\omega_{c}s+\omega_{c}^{2}}$$ (1)

Where $\omega_{c}=2\pi f_{c}$ is the cutoff angular frequency. This filter has a maximally flat passband frequency response, effectively removing high-frequency noise (e.g., small device vibrations, transient jitters) while preserving low-frequency signals representing major cow behavior patterns, such as the rhythmic chewing during rumination and the periodic head movements during feeding.

*Computation of resultant acceleration:* To further extract features directly related to motion intensity and provide a direction-independent measure of overall activity, the resultant acceleration (RA) is calculated for each sample using Equation 2:

$$A_{\mathrm{vm}}(t)=\sqrt{A_{x}{\left(t\right)}^{2}+A_{y}{\left(t\right)}^{2}+A_{z}{\left(t\right)}^{2}}$$ (2)

Where Ax(t), Ay(t), Az(t) denote the filtered acceleration values along the X, Y, and Z axes at time *t*.

### 2.4 Time series to image conversion

Although IMU-based methods typically rely on 1D time-series modeling, such representations may not fully capture long-range temporal dependencies and complex correlations between signal segments. To address this limitation, GADF is introduced to transform the time series into a two-dimensional representation by encoding pairwise angular relationships between time points. This process preserves temporal dependencies in a structured form and converts them into spatial patterns, enabling convolutional neural networks to effectively learn both local variations and global temporal structures. Given a preprocessed and segmented single-channel acceleration sequence of length *n*, $X=\{x_{1},x_{2},\ldots ,x_{n}\}$ , the conversion to a GADF image proceeds as follows:

Normalization: First, each value in the sequence *X* is normalized to the interval [−1, 1] to mitigate the effects of differing sensor units and individual activity intensity variations. The formula is shown in Equation 3:


$${\tilde{x}}_{i}=\frac{x_{i}-\min(X)}{\max(X)-\min(X)}$$ (3)

The normalized sequence is denoted as $\tilde{X}=\{{\tilde{x}}_{1},{\tilde{x}}_{2},\ldots ,{\tilde{x}}_{n}\}$ .

Polar Coordinate Transformation: The normalized time series values are mapped onto a polar coordinate system. Specifically, each value ${\tilde{x}}_{i}$ is interpreted as an angle (via its cosine), and its corresponding time index *i* as a radius. The angle $\phi_{i}$ is computed using the arccosine function as shown in Equation 4:


$$\phi_{i}=\arccos({\tilde{x}}_{i}),\;{\tilde{x}}_{i}\in [-1,1],\;\phi_{i}\in [0,\pi ]$$ (4)

The radius $r_{i}$ is defined proportionally to the timestamp, here simply mapped as Equation 5:


$$r_{i}=\frac{i}{n}$$ (5)

Thus, each time point *i* is mapped to a point $\left(\phi_{i},r_{i}\right)$ in the polar coordinate system.

Construction of the Gramian Angular Difference Field (GADF): The essence of GADF is to construct a Gramian matrix by computing the angular differences (sine values) between pairs of polar coordinate points. The calculation formula is shown in Equation 6:


$$\text{GADF}=[\begin{array}{ccc} \sin(\phi_{1}-\phi_{1}) & \cdots & \sin(\phi_{1}-\phi_{n})\\ \vdots & \ddots & \vdots \\ \sin(\phi_{n}-\phi_{1}) & \cdots & \sin(\phi_{n}-\phi_{n}) \end{array}]={\sqrt{I-{\tilde{X}}^{2}}}^{T}\cdot \tilde{X}-{\tilde{X}}^{T}\cdot \sqrt{I-{\tilde{X}}^{2}}$$ (6)

Where $\tilde{X}$ is the row vector of the normalized sequence, and *I* is a unit vector. This formula utilizes the trigonometric identity $\sin(\phi_{i}-\phi_{j})=\sin\phi_{i}\cos\phi_{j}-\cos\phi_{i}\sin\phi_{j}$ .

Grayscale Image Generation and Pseudo-color Encoding: The generated GADF is a 64 × 64 real symmetric matrix, with each element’s value range being [−1, 1]. To enhance the visual distinguishability of the images and potentially provide richer low-level features for the convolutional neural network, we apply pseudo-color mapping to the grayscale GADF images. Specifically, colormaps are employed to convert the single grayscale intensity values into three-dimensional RGB color vectors. Consequently, each original resultant acceleration sequence is ultimately transformed into a 64 × 64 pixel pseudo-color image.

### 2.5 Global–local feature extraction network

We designed a feature extraction network that integrates both global and local representations. The proposed architecture, named GFENet, consists of three main components: a global feature extraction branch, a local feature extraction branch, and a Multi-Head Cross-Attention Coupling (MCC) module. In this study, ResNet18 is adopted as the backbone network for global feature extraction. The choice is motivated by its effective trade-off between model complexity and feature representation capability. Compared with deeper networks such as ResNet50 or DenseNet, ResNet18 contains fewer parameters and lower computational cost, which is advantageous for efficient training and potential deployment in resource-constrained wearable sensing systems. The global branch incorporates dilated convolution modules to capture broad behavioral patterns and overall motion trends. The local branch employs region selection modules to focus on fine-grained details and specific motion signatures within the images. Features extracted from both branches are then fused through a multi-head cross-attention mechanism, which enables effective interaction between global context and local details. The total number of parameters of the proposed GFENet is approximately 14.6 M, which is comparable to ResNet18-based architectures while introducing additional global–local interaction modules.

#### 2.5.1 Global feature extraction branch

**Figure 3:**
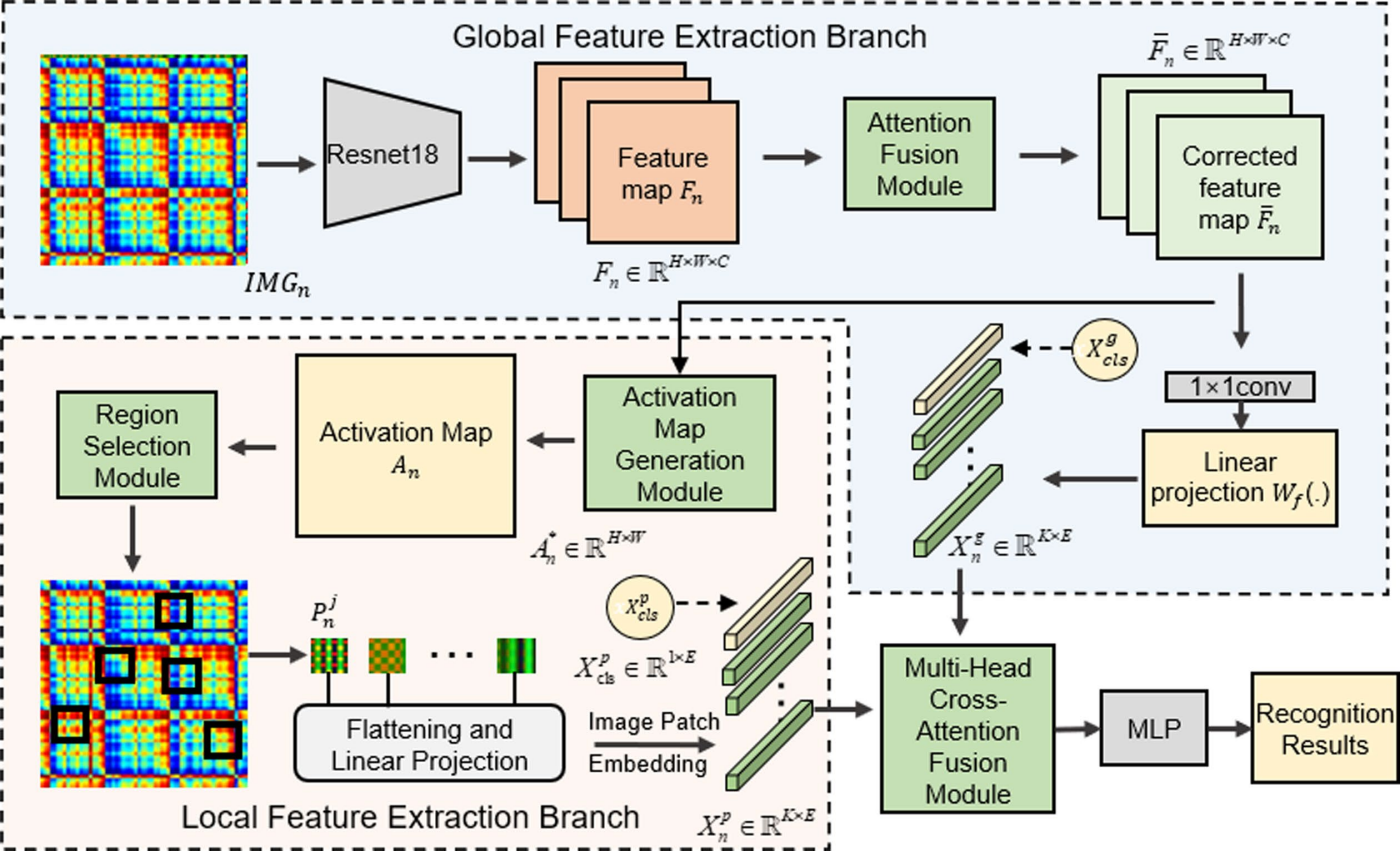
Structure of the dual-stream feature enhancement model.

As illustrated in Figure 3, let IMGₙ denote the *n-th* training image. After passing through the five convolutional blocks of the ResNet18 network, a feature map $F_{n}\in {\mathbb{R}}^{H\times W\times C}$ is obtained, where *H*, *W*, and *C* represent the height, width, and number of channels, respectively. This feature map is then enhanced via an attention fusion module to generate a refined feature map ${\bar{F}}_{n}\in {\mathbb{R}}^{H\times W\times C}$ . Subsequently, a 1 × 1 convolution followed by a linear projection is applied to derive the global feature representation $X_{n}^{g}\in {\mathbb{R}}^{K\times E}$ , where *K* denotes the number of information units and *E* the embedding dimension.

**Figure 4:**
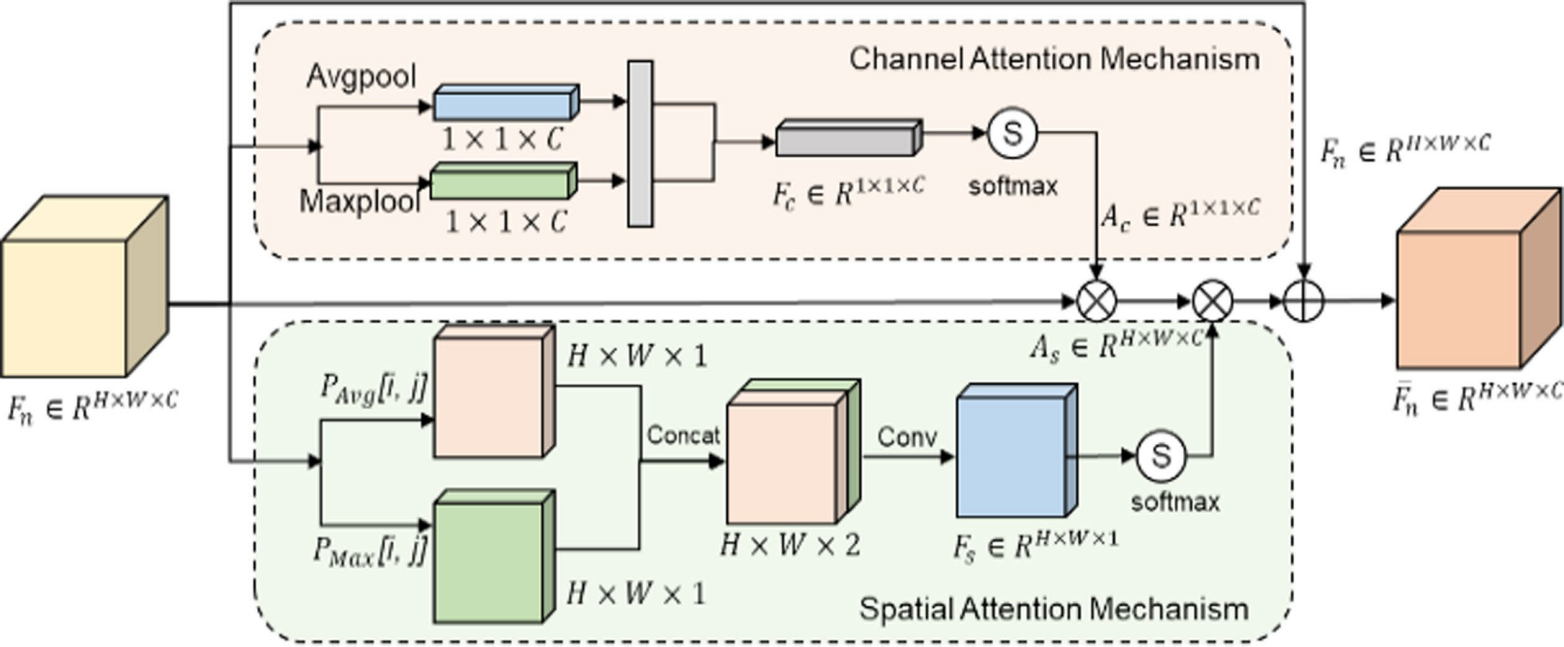
Architecture of the attention fusion module.

Attention Fusion Module: To guide the convolutional neural network (CNN) to focus on significant channels and critical regions within the feature maps, an attention fusion module is proposed—drawing inspiration from SENet and CBAM—and integrated after the fifth convolutional block of ResNet18. This module enhances the global feature extraction capability by jointly modeling channel-wise and spatial attention, as shown in Figure 4.

Let $F_{n}\in {\mathbb{R}}^{H\times W\times C}$ denote the input feature map extracted by ResNet18. First, $F_{n}$ is separately processed by the channel attention and spatial attention submodules, resulting in a channel attention map $A^{c}\in {\mathbb{R}}^{1\times 1\times C}$ and a spatial attention map $A^{s}\in {\mathbb{R}}^{H\times W\times 1}$ , respectively. The refined feature map is then obtained by sequentially applying both attention maps to $F_{n}$ , followed by a residual connection with the original feature, as described in Equation 7:


$${\bar{F}}_{n}=F_{n}+\sigma (A^{s}⊗\sigma (A^{c}⊗F_{n}))\;$$ (7)

Here, σ denotes the Softmax function, and ⊗ represents the element-wise multiplication operation. This operation adaptively refines feature representations by emphasizing informative responses while preserving original feature integrity.

Channel Attention Submodule: This module aims to capture informative channels by exploiting global spatial information. Specifically, both global average pooling (GAP) and global max pooling (GMP) are performed on feature map, followed by shared fully connected (FC) layers. The resulting outputs are summed to yield the channel attention map $A^{c}\in {\mathbb{R}}^{1\times 1\times C}$ , as formulated in Equation 8:


$$A^{c}=\mathrm{FC}(\mathrm{GAP}(F_{n}))+\mathrm{FC}(\mathrm{GMP}(F_{n}))$$ (8)

Spatial Attention Submodule: To highlight the crucial spatial regions, this submodule first computes the channel-wise average and maximum across all feature maps, yielding two spatial descriptors $P^{\mathrm{Avg}}$ and $P^{\max}$ . These are concatenated and passed through a convolutional layer with a 7×7 kernel to generate the spatial attention map $A^{s}\in {\mathbb{R}}^{H\times W\times 1}$ , as given in Equation 9:


$$A^{s}={\text{Conv}}_{7\times 7}(\text{Concat}(P^{\mathrm{Avg}},P^{\max}))$$ (9)

Global Feature Generation: To reduce data dimensionality and obtain fixed-dimensional global features conducive to subsequent feature coupling, a 1×1 convolution is first applied to the attention-enhanced feature map ${\bar{F}}_{n}\in {\mathbb{R}}^{H\times W\times C}$ , reducing the number of channels to *K*. Then, the result is flattened along the channel dimension and mapped via a linear projection matrix $W_{F}$ to generate the global feature tokens, denoted as $X_{n}^{g}\in {\mathbb{R}}^{K\times E}$ . The process can be formulated as Equation 10:


$$X_{n}^{g}=W_{F}[\text{Flatten}({\text{Conv}}_{1\times 1}({\bar{F}}_{n}))]$$ (10)

Where ${\text{Conv}}_{1\times 1}()$ represents the 1×1 convolution operation, Flatten() denotes channel-wise flattening, and $W_{F}[]$ denotes linear projection. This process converts convolutional feature maps into compact global tokens suitable for subsequent cross-attention fusion. To facilitate the subsequent multi-head cross-attention feature fusion, a learnable global class token $X_{\mathrm{cls}}^{g}\in {\mathbb{R}}^{1\times E}$ is prepended to the global feature tokens. The final global feature representation becomes: $\left\{X_{\mathrm{cls}}^{g},X_{n}^{g}\right\}$ .

#### 2.5.2 Local feature extraction branch

As illustrated in Figure 3, let ${\mathrm{IMG}}_{n}$ denote the *n-th* training image. To extract its local features, a cropping module is designed under the guidance of an attention activation map to obtain image regions potentially containing key information, which are subsequently embedded to generate local features. The detailed procedures of this branch are as follows:

Activation Map Generation Module: Critical local regions often occupy a small area and can be easily obscured by background noise. To enable accurate extraction of fine-grained features, an Attention Activation Map Generation (AMG) module is designed inspired by the SCDA method in image retrieval. In this module, the attention activation map $A_{n}$ is obtained by summing the values at each spatial location of the enhanced feature map across the channel dimension.

Specifically, for each spatial location (i,j) in the feature map ${\bar{F}}_{n}\in {\mathbb{R}}^{H\times W\times C}$ , the attention activation value is calculated as using Equation 11:


$$A_{n}(i,j)=\sum_{k=1}^{C}{\bar{F}}_{n}(i,,j,,k),\;\;\;\;(1\leq i\leq W,1\leq j\leq H)$$ (11)

This generates a spatial map highlighting regions with strong motion-related responses in the encoded image. The resulting activation map is normalized by Equation 12:


$$A_{n}^{*}=\frac{A_{n}-\min(A_{n})}{\max(A_{n})-\min(A_{n})}$$ (12)

This normalization ensures that activation values are comparable across different samples.

Finally, $A_{n}^{*}\in {\mathbb{R}}^{H\times W}$ is upsampled to the original image resolution via bilinear interpolation, yielding the final attention activation map $A_{n}$ used to guide local region extraction.

Region Selection Module: Observing the attention map reveals that regions with higher activation values correspond to areas rich in semantic content. In the context of GADF-transformed IMU signals, these high-response regions correspond to specific temporal segments exhibiting distinctive motion patterns, such as strong chewing cycles, rhythmic rumination phases, or transitions between different behaviors. Based on this, a region selection module is designed using Non-Maximum Suppression (NMS) to select the top *T* image regions with the highest response values from the original image. The detailed procedure of the region selection module is as follows: a sliding window of fixed size scans the attention map from top to bottom and left to right. For each window $A_{p}$ , the mean response $\mu_{p}$ is computed as Equation 13:


$$\mu_{p}=\frac{1}{H_{p}W_{p}}\sum_{i=0}^{W_{p}-1}\sum_{j=0}^{H_{p}-1}A_{p}(i,j)$$ (13)

Where $H_{p}$ and $W_{p}$ represent the height and width of the sliding window, respectively. To avoid excessive overlap among regions and ensure diverse local features, NMS is applied to select the top *T* windows with the highest responses. These correspond to the cropped regions $\left\{P_{n}^{j}|\;j=1,2,\ldots ,T\right\}$ , where $P_{n}^{j}$ denotes the *j-th* selected region from the *n-th* image. Each of these *T* regions $\left\{P_{n}^{j}|\;j=1,2,\ldots ,T\right\}$ is then flattened and embedded to obtain local tokens $X_{n}^{p}\in {\mathbb{R}}^{K\times E}$ with the same shape as the global features $X_{n}^{g}$ . A local class token $X_{\mathrm{cls}}^{p}\in {\mathbb{R}}^{1\times E}$ is also introduced for subsequent cross-attention operations. The final local feature representation is: $\left\{X_{\mathrm{cls}}^{p},X_{n}^{p}\right\}$ .

#### 2.5.3 Multi-head cross-attention coupling (MCC) module

To enhance the interaction between global and local features, inspired by CrossViT, we design a Multi-Head Cross-Attention Coupling (MCC) module by stacking L multi-head cross-attention encoder layers. Unlike CrossViT, which adopts symmetric and bidirectional feature exchange between dual branches, the proposed MCC introduces a lightweight unidirectional coupling strategy, where the local class token serves as the Query to selectively aggregate discriminative information from global representations. This design reduces redundant cross-branch interactions while improving task-specific feature refinement for fine-grained behavior discrimination.

**Figure 5:**
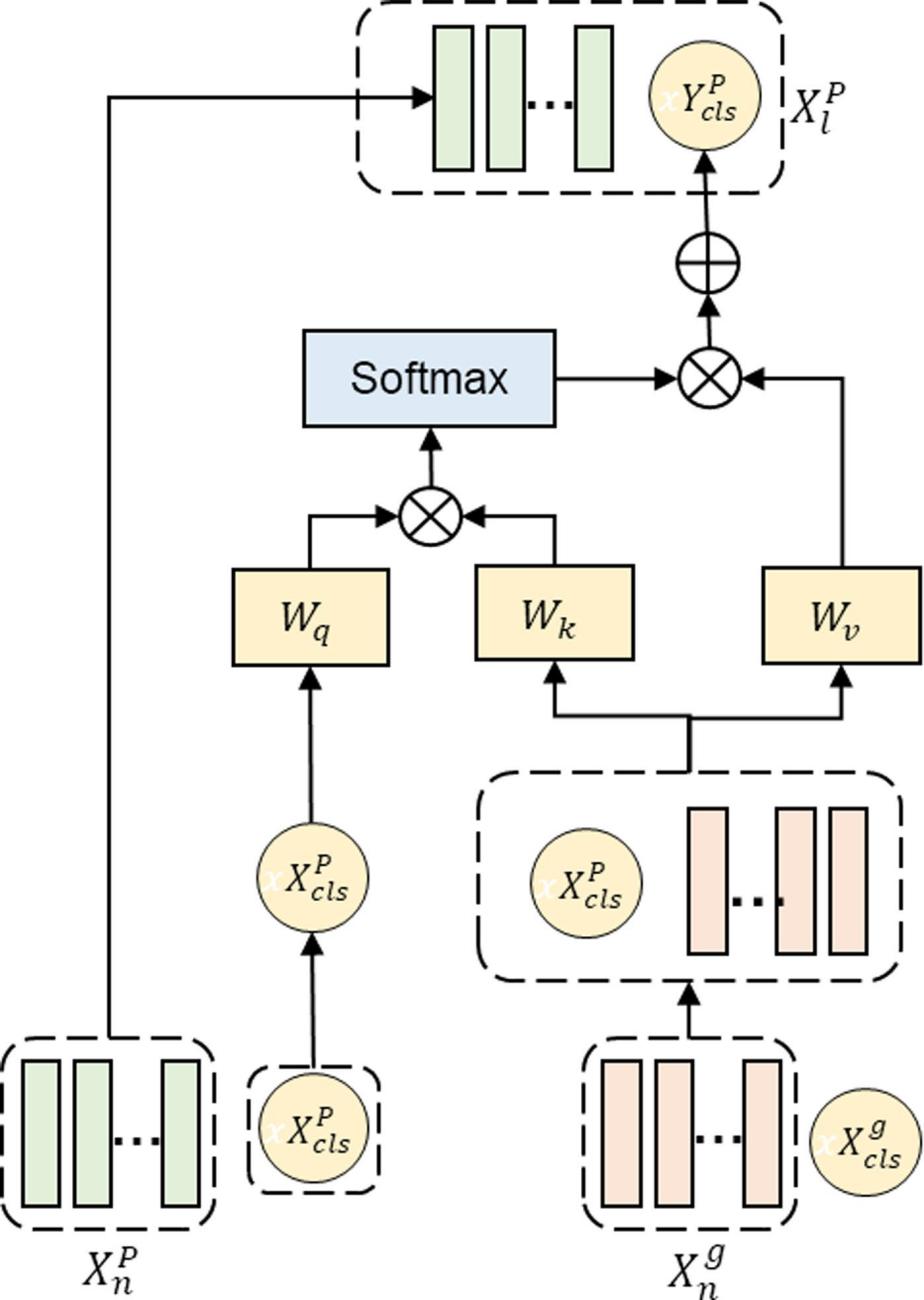
Multi-head cross-attention coupling module.

After processing by the methods described, the global feature $\left\{X_{\mathrm{cls}}^{g},X_{n}^{g}\right\}$ and the local feature $\left\{X_{\mathrm{cls}}^{p},X_{n}^{p}\right\}$ are obtained. To couple the global information into the local features, a single-direction multi-head cross-attention fusion process is illustrated in Figure 5. The detailed procedure is as follows:

First, the local class token $X_{\mathrm{cls}}^{p}$ and the global feature $X_{n}^{g}$ are concatenated, as shown in Equation 14:


$$X=\text{Concat}(X_{\mathrm{cls}}^{p},X_{n}^{g})$$ (14)

Next, the cross-attention is computed between $X_{\mathrm{cls}}^{p}$ and $X_{n}^{g}$ , where the local class token $X_{\mathrm{cls}}^{p}$ serves as the sole Query, integrating information contained in the global feature $X_{n}^{g}$ into $X_{\mathrm{cls}}^{p}$ . The cross-attention calculation is expressed by Equation 15:


$$\left\{ \begin{array}{l} q=W_{q}X_{\mathrm{cls}}^{p},\;\;\;\;k=W_{k}X_{n}^{g},\;\;\;\;v=W_{v}X_{n}^{g}\\ A=\text{softmax}(\frac{qk^{T}}{\sqrt{D/h}})\\ X_{\mathrm{CA}}=\mathrm{Av} \end{array}\right.$$ (15)

Where $W_{q}$ , $W_{k}$ , $W_{v}\in {\mathbb{R}}^{D\times (D/h)}$ represent the projection matrices for Query, Key, and Value respectively, and *D* and *h* denote the embedding dimension and the number of attention heads. Following the multi-head attention mechanism, multiple attention heads are employed to improve cross-attention, forming a multi-head cross-attention module.

Furthermore, the cross-attention coupling module incorporates layer normalization and residual connections. The overall computation of the multi-head cross-attention coupling encoder is given by Equation 16:


$$\left\{ \begin{array}{l} Y_{\mathrm{cls}}^{p}=X_{\mathrm{cls}}^{p}+\mathrm{MCA}(\mathrm{LN}(X_{\mathrm{cls}}^{p}),\mathrm{LN}(X_{n}^{g}))\\ Y_{p}^{l}=\text{Concat}(Y_{\mathrm{cls}}^{p},X_{n}^{p}) \end{array}\right.$$ (16)

Where LN () denotes layer normalization, MCA () represents the multi-head cross-attention module, $Y_{\mathrm{cls}}^{p}$ is the updated local class token after correction, and $Y_{p}^{l}$ is the concatenated input to the next layer of the cross-attention encoder. After multiple layers of the cross-attention encoder, the model obtains rich interaction information between the global and local branches, with complementary features fused in their respective class tokens. Finally, the updated global and local class tokens are concatenated and fed into a multi-layer perceptron (MLP) classification head to output the final classification result.

### 2.6 Performance evaluation metrics

To evaluate the performance of the proposed method, four commonly used metrics were adopted, including accuracy, precision, recall, and F1-score. In addition, a confusion matrix was used to analyze the classification results for each behavior category.

For a multi-class classification problem with *K* classes, the confusion matrix $C\in {\mathbb{R}}^{K\times K}$ is defined as $C=[C_{i,j}]$ , where each element $C_{i,j}$ represents the number of samples whose true class is *i* and are predicted as class *j*. The diagonal elements represent correctly classified samples, while off-diagonal elements represent misclassifications.

Based on the confusion matrix, the evaluation metrics are defined as follows Equations 17–20:


$$\text{Accuracy}=\frac{\sum_{k}C_{k,k}}{\sum_{i}\sum_{j}C_{i,j}}$$ (17)

$$\text{Precision}=\frac{\mathrm{TP}}{\mathrm{TP}+\mathrm{FP}}$$ (18)

$$\text{Recall}=\frac{\mathrm{TP}}{\mathrm{TP}+\mathrm{FN}}$$ (19)

$$F1=\frac{2\cdot \text{Precision}\cdot \text{Recall}}{\text{Precision}+\text{Recall}}$$ (20)

The confusion matrix provides a detailed view of misclassification between different behaviors (feeding, rumination, and movement), while these metrics give an overall evaluation of model performance.

## 3 Results

### 3.1 Dataset description

The dataset used in this study was collected from an 11-day on-site experiment conducted with seven Holstein cows in a typical farm environment in Inner Mongolia, China. Approximately 64 h of valid synchronized data were obtained, resulting in a total of 1,252,536 raw tri-axial acceleration samples (sampling frequency: 10 Hz). The collected and annotated cow behavior data consist of seven columns: ‘date’, ‘time’, ‘cow_num’, ‘acc_x’, ‘acc_y’, ‘acc_z’, and ‘behavior’. These columns represent the date, time, numerical identifier of the cow, tri-axial acceleration measurements, and the corresponding behavior label, respectively.

**Figure 6:**
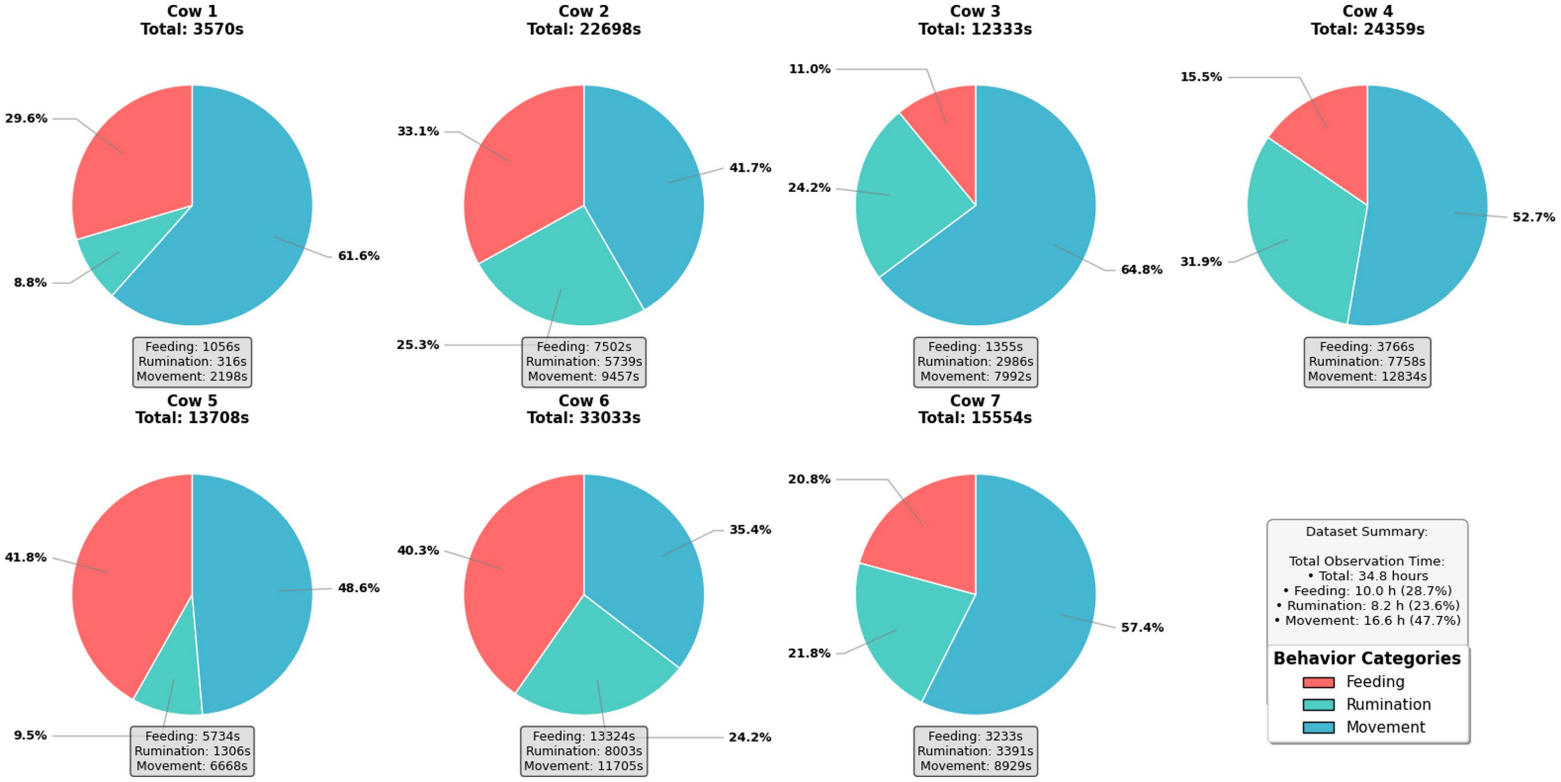
Time distribution of data collection for individual cows.

Based on manual annotations from synchronized video recordings, the collected data were categorized into three core behaviors: feeding, rumination, and moving. The behavior duration statistics for each cow are illustrated in Figure 6. After applying a 6.4-s sliding window segmentation and preprocessing, a total of 18,132 valid samples were obtained for model training and evaluation.

Table 1 presents the temporal distribution of the raw data for the three behaviors. It can be observed that moving accounts for the largest portion of data, while feeding and rumination are relatively balanced and consistent with the typical daily behavior patterns of cows. This distribution provides a solid foundation for constructing a balanced classification model.

**Table 1:** Temporal distribution statistics of the cow behavior dataset.

Behavior category	Total duration (s)	Proportion (%)	Number of samples
Feeding	35,977.1	28.9	5,421
Rumination	29,498.5	23.7	4,146
Moving	59,264.5	47.5	8,565
Total	124,740.1	100.0	18,132

To ensure objective evaluation and model generalization, an individual-based data splitting strategy was adopted. All data from cow #7 were designated as an independent validation set, as its behavior data were the most evenly distributed among the three categories (approximately 1:1:2), making it highly representative. The remaining data from the other six cows were randomly divided into a training set and a test set, with a ratio of approximately 8:2.

### 3.2 Image conversion results and visual analysis

**Figure 7:**
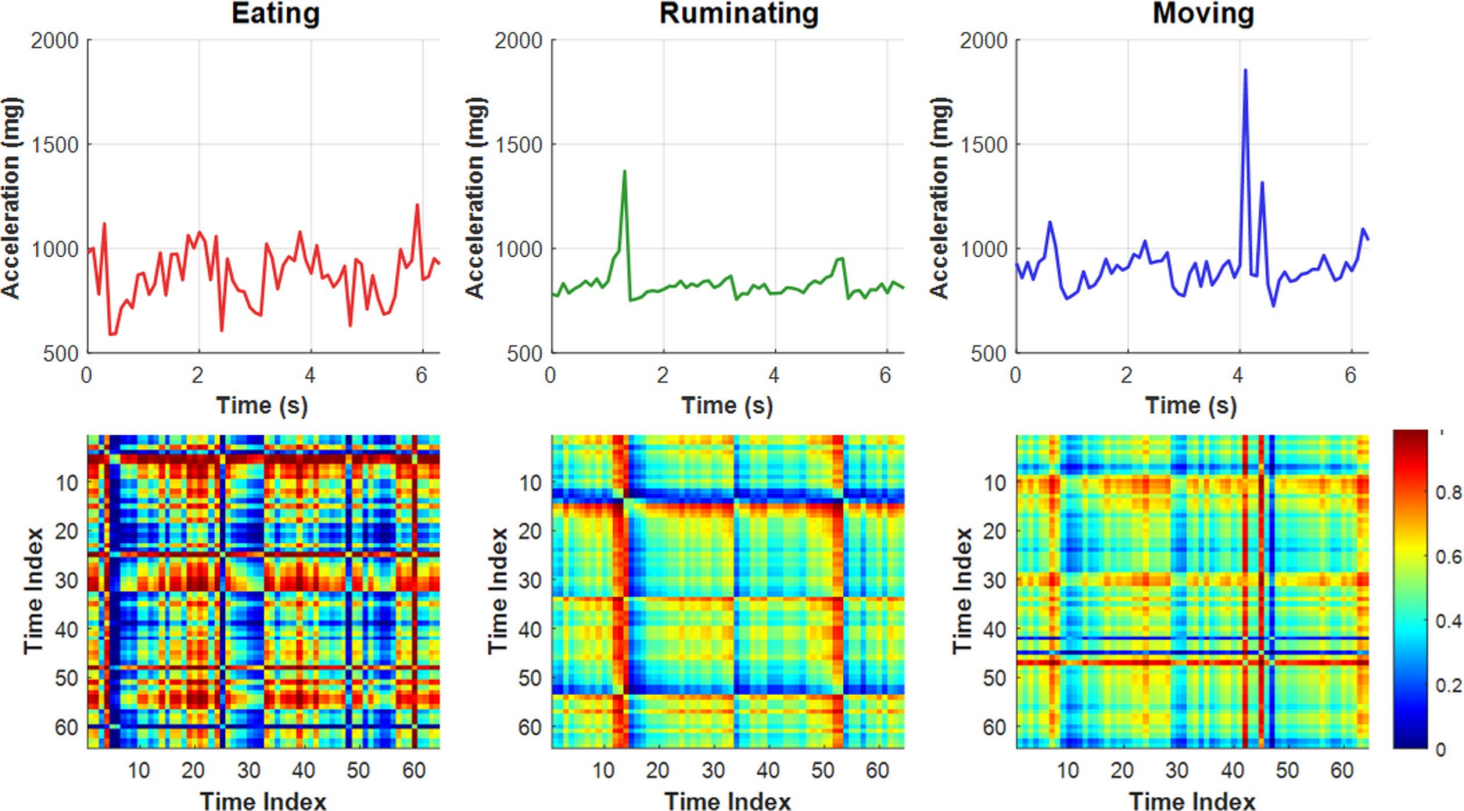
Raw acceleration signals and corresponding converted images for different behavior categories.

Figure 7 presents representative examples of the raw acceleration signals and their corresponding encoded images for three cow behavior categories: feeding, rumination, and moving. The first row shows the resultant acceleration signals within a typical 6.4 s window, while the second row displays the corresponding time-series images generated by the proposed encoding method. Distinct differences can be observed among the three behaviors in both the temporal domain and the image domain.

For the feeding behavior, the acceleration signal exhibits moderate-amplitude fluctuations with relatively frequent variations, reflecting continuous head and jaw movements during feed intake. In the encoded image, these characteristics are manifested as dense and repetitive texture patterns distributed across the time–time plane, indicating strong short-term temporal correlations and repetitive motion dynamics.

The rumination behavior is characterized by a more stable and rhythmic acceleration pattern, with lower overall amplitude and occasional pronounced peaks corresponding to cud chewing cycles. The converted image shows more regular and structured patterns, with smoother transitions and clearer repetitive bands, reflecting the quasi-periodic and consistent nature of rumination-related movements.

In contrast, the moving behavior displays markedly different characteristics. The acceleration signal contains higher-amplitude variations and abrupt peaks caused by body displacement, stepping motions, and head movements. Accordingly, the encoded image exhibits more irregular and fragmented structures, with stronger contrast variations and disrupted textures, highlighting the non-stationary and high-intensity nature of moving behavior.

The image representations effectively amplify the intrinsic differences among feeding, rumination, and moving behaviors by transforming one-dimensional temporal signals into two-dimensional structural patterns. These visually distinguishable characteristics provide rich discriminative information for subsequent feature extraction and classification, demonstrating the effectiveness of the proposed time-series image conversion strategy.

### 3.3 Recognition results

**Figure 8:**
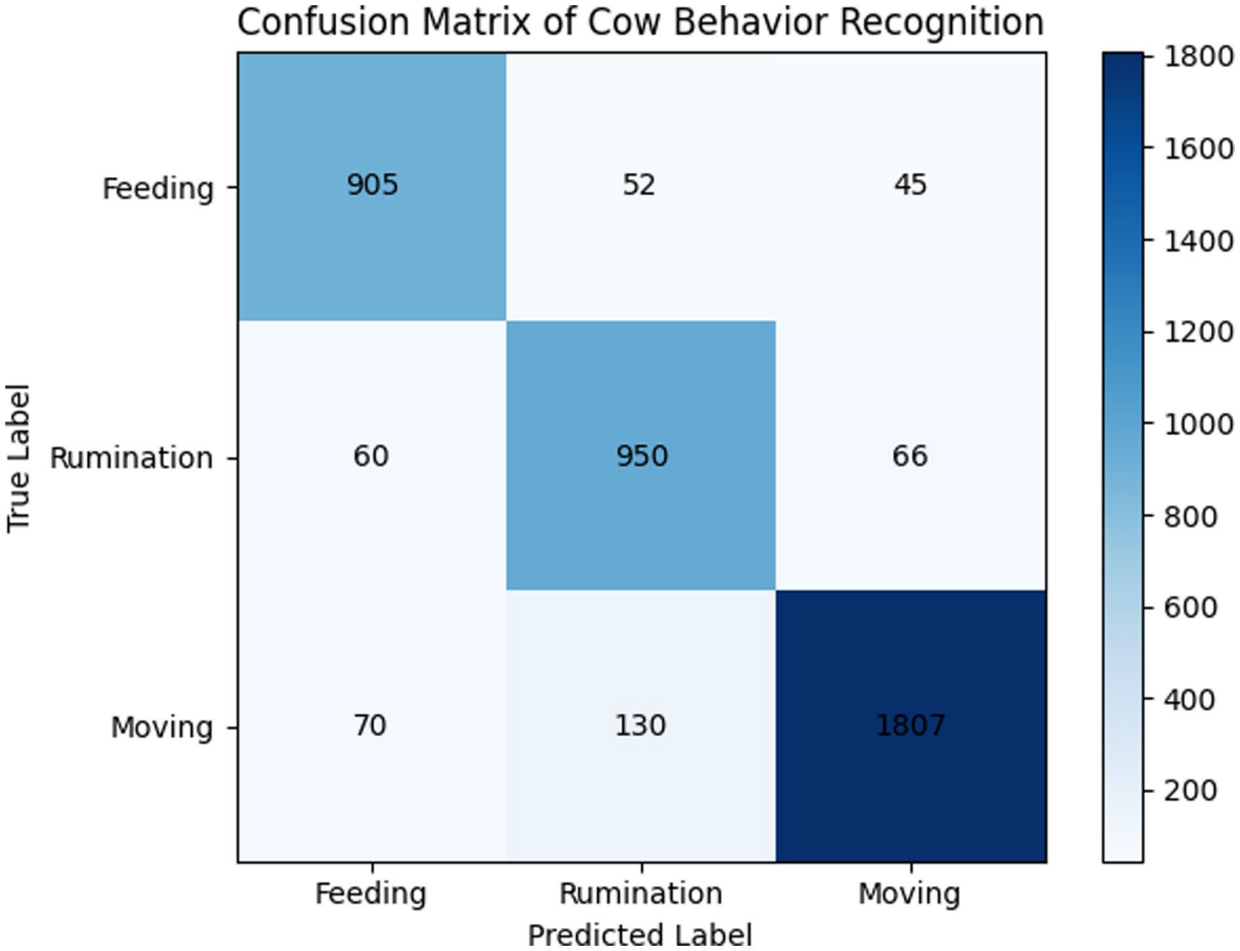
Confusion matrix of the proposed method on the test dataset.

To evaluate the performance of the proposed cow behavior recognition framework, classification results on the test set are analyzed in terms of per-class precision, recall, F1-score, and overall accuracy. Table 2 shows the recognition performance for different behavior categories. All experiments were independently repeated five times using different random seeds to reduce randomness and ensure result reliability. Figure 8 illustrates the confusion matrix of the proposed method on the test dataset. The numerical values in the matrix strictly correspond to the sample counts of each behavior category.

**Table 2:** Recognition performance for different behavior categories.

Behavior category	Precision (%)	Recall (%)	F1-score (%)
Feeding	87.44 ± 0.52	90.32 ± 0.41	88.86 ± 0.46
Rumination	83.92 ± 0.63	88.29 ± 0.57	86.05 ± 0.55
Moving	94.21 ± 0.38	90.03 ± 0.44	92.08 ± 0.40

As shown in Table 2 and Figure 8, the proposed method achieves robust recognition performance across all three behavior categories. Overall, the proposed method achieves an overall recognition accuracy of 90.12% on the test set, demonstrating stable and reliable classification performance across different cow behavior categories under real farm conditions. The feeding behavior exhibits a high recall of 90.32%, indicating that chewing-related motion patterns captured by the inertial signals are effectively recognized. The rumination behavior shows slightly lower precision and F1-score, which can be attributed to partial motion similarity with feeding behaviors, especially during subtle jaw movements. Nevertheless, the model maintains stable discrimination capability with an F1-score exceeding 86%. The moving behavior achieves the highest precision (94.21%) and F1-score (92.08%), reflecting the strong distinguishability of large-amplitude and non-stationary motion patterns associated with body displacement and head movements.

From the confusion matrix, for the feeding category, 905 out of 1,002 samples are correctly classified, while 52 samples (5.19%) are misclassified as rumination and 45 samples (4.49%) are misclassified as moving. For the rumination category, 950 out of 1,076 samples are correctly identified, with 60 samples (5.58%) misclassified as feeding and 66 samples (6.13%) misclassified as moving. For the moving category, 1,807 out of 2,007 samples are correctly classified, while 130 samples (6.48%) are misclassified as rumination and 70 samples (3.49%) are misclassified as feeding.

### 3.4 Effect of different time window lengths

To investigate the influence of time window length on behavior recognition performance, experiments were conducted using five different window sizes: 32, 48, 64, 96, and 128 samples. As shown in Table 3, when the window is too short (32 samples), limited temporal context leads to lower performance. Increasing the window size to 48 and 64 samples significantly improves performance, with 64 samples achieving the best results. This indicates that a moderate-length window can better capture complete behavioral patterns such as chewing and rumination cycles. When the window is further increased to 96 and 128 samples, performance slightly decreases, likely due to redundant or less relevant temporal information. To further validate these observations, a statistical significance analysis (one-way ANOVA) was performed across all window settings, confirming that the differences among configurations are statistically significant (*p* < 0.05). In particular, the 64-sample window consistently outperforms other settings. Therefore, 64 samples (6.4 s at 10 Hz) is selected as the optimal window size for subsequent experiments, as it provides the best balance between temporal completeness and feature discriminability.

**Table 3:** Performance evaluation of the proposed method under different time window lengths.

Window size	Accuracy (%)	Precision (%)	Recall (%)	F1-score (%)
32	87.84 ± 0.58	87.12 ± 0.63	86.95 ± 0.60	87.03 ± 0.59
48	88.72 ± 0.50	88.40 ± 0.52	88.10 ± 0.55	88.25 ± 0.53
64	90.12 ± 0.42	90.54 ± 0.38	90.01 ± 0.41	90.27 ± 0.40
96	89.35 ± 0.47	89.70 ± 0.44	89.10 ± 0.48	89.40 ± 0.46
128	88.96 ± 0.55	89.18 ± 0.50	88.42 ± 0.57	88.80 ± 0.53

### 3.5 Ablation study

To evaluate the contribution of each component in the proposed model, a series of ablation experiments were conducted by progressively removing or modifying key modules. Ablation study is a commonly used strategy to analyze the effectiveness of individual components by isolating their contributions to the final performance. The following model variants were designed for comparison:

*B1*: Full model (GFENet), which integrates all proposed components.

*B2*: Global branch only, corresponding to the Global Feature Extraction Branch described in Section 2.5.1.

*B3*: Local branch only, corresponding to the Local Feature Extraction Branch described in Section 2.5.2.

*B4*: Global and local branches with simple feature concatenation, instead of the proposed MCC module described in Section 2.5.3.

*B5*: Baseline model, which only retains the basic ResNet backbone without any proposed enhancement modules.

**Figure 9:**
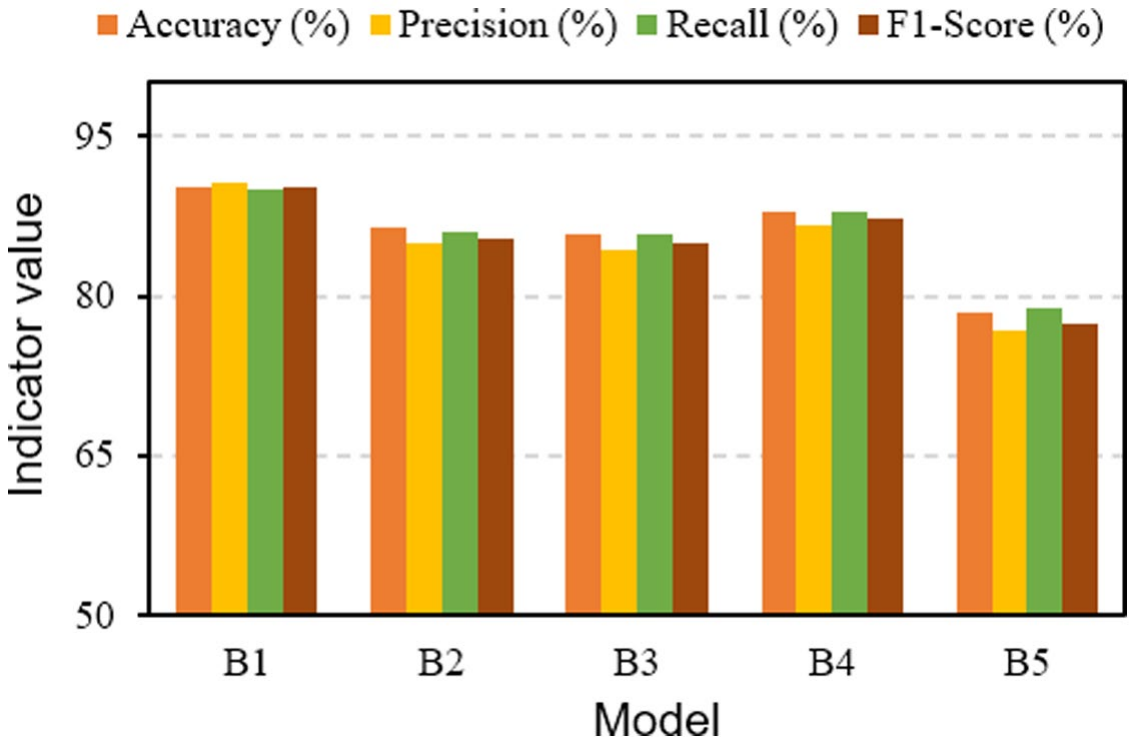
Performance comparison of different network configurations. B1: Full model; B2: Global branch only; B3: Local branch only; B4: Global and local branches with simple feature concatenation instead of MCC; B5: Baseline model.

**Figure 10:**
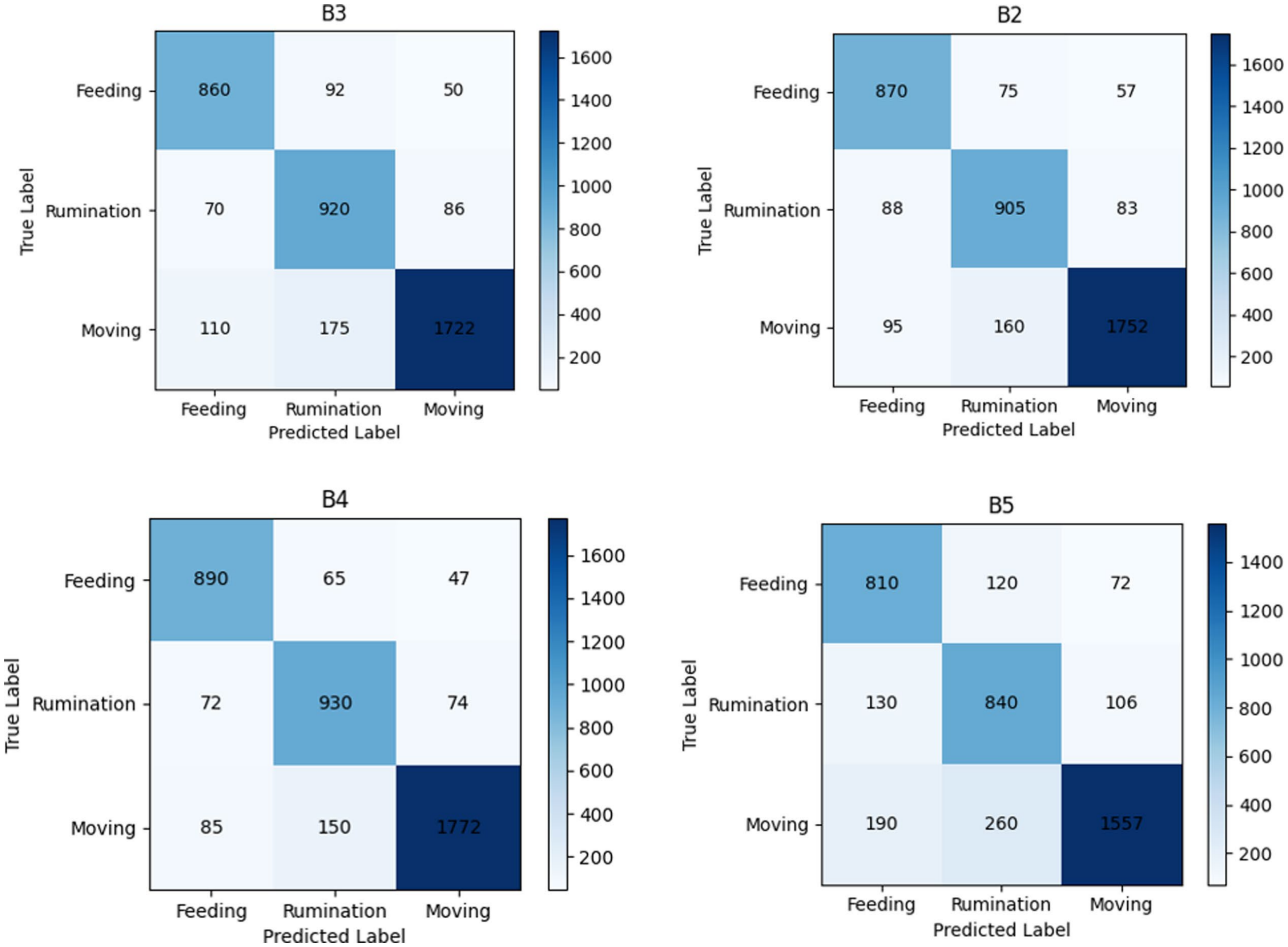
Confusion matrices of different network configurations.

Figure 9 reports the quantitative performance of different network configurations, while Figure 10 illustrates the corresponding confusion matrices.

The full model (B1), which integrates global enhancement, local attention, and multi-head cross-attention coupling, achieves the best overall performance, reaching an accuracy of 89.65% and a macro-averaged F1-score of 88.99%. When only the global enhancement branch (B2) or the local attention branch (B3) is retained, the recognition performance decreases noticeably, indicating that both branches capture complementary behavioral features. Replacing the MCC module with simple feature concatenation (B4) also leads to performance degradation, demonstrating the effectiveness of structured global–local feature interaction. The baseline ResNet model (B5) yields the lowest accuracy and F1-score, with more severe misclassifications among feeding, rumination, and moving behaviors, as observed in Figure 10. These results confirm that each proposed component contributes positively to the final recognition performance, and their joint integration is essential for accurate cow behavior recognition.

To further evaluate the effectiveness of the proposed GADF representation, we conduct comparative experiments using both raw IMU signals and GADF-based image representations. In this experiment, we adopt a set of standard CNN backbones for feature extraction. Importantly, these networks are different from the proposed GFENet architecture and are used only as independent baseline models without any additional components such as global–local branches or cross-attention fusion modules. Therefore, the models used in this experiment consist solely of backbone networks for feature learning.

For a fair comparison, corresponding 1D and 2D versions of each backbone are constructed. Specifically, 1D CNN variants (e.g., 1D ResNet, 1D VGG, 1D DenseNet, and 1D MobileNet) are applied to raw data, while their 2D counterparts (e.g., 2D ResNet, 2D VGG, 2D DenseNet, and 2D MobileNet) are applied to GADF image representations. In this way, the network architecture within each pair remains consistent, and only the input representation is changed.

**Figure 11:**
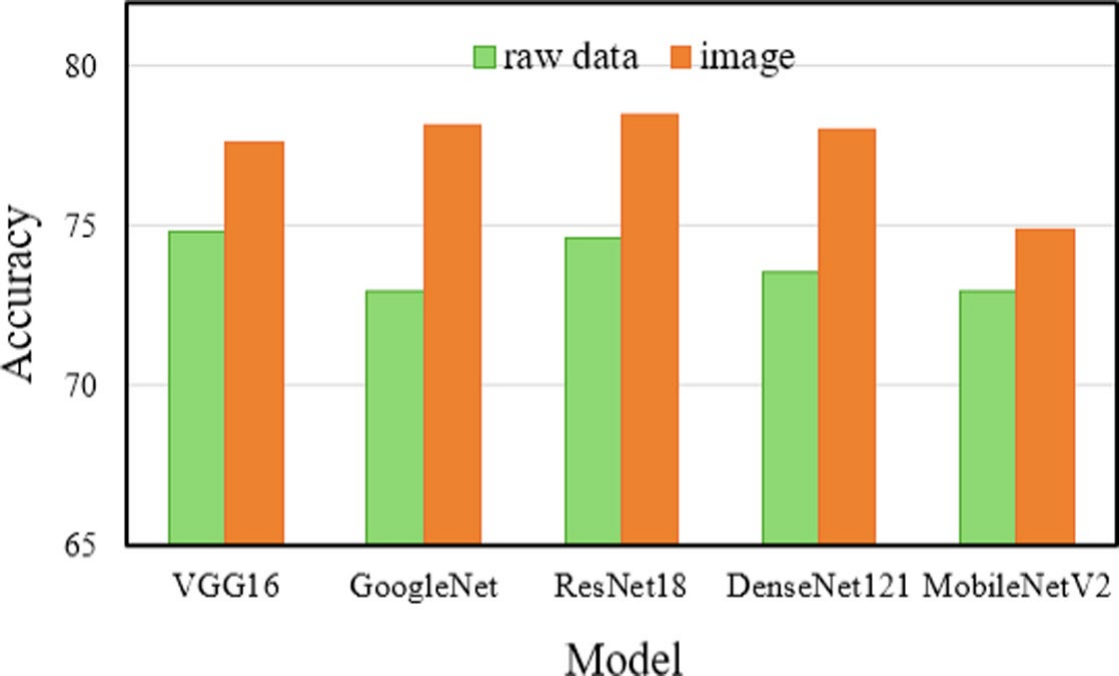
Performance comparison of different CNN models on raw IMU data and image-based (GADF) representation.

As shown in Figure 11, all paired models consistently achieve better performance when using GADF-based inputs compared to raw IMU signals. This indicates that the improvement is primarily attributed to the proposed time-series-to-image transformation rather than differences in network design. The GADF representation effectively converts temporal dependencies into structured 2D patterns, enabling CNNs to better capture local spatial correlations and discriminative texture information. Among all backbone models, ResNet18 achieves the best or comparable performance under both input settings, reaching an accuracy of 78.51% in the GADF-based case. This further demonstrates the stability and effectiveness of residual learning in processing both 1D and 2D representations. Overall, these results validate the general effectiveness of the proposed GADF representation under a strictly controlled and independent baseline comparison setting.

**Figure 12:**
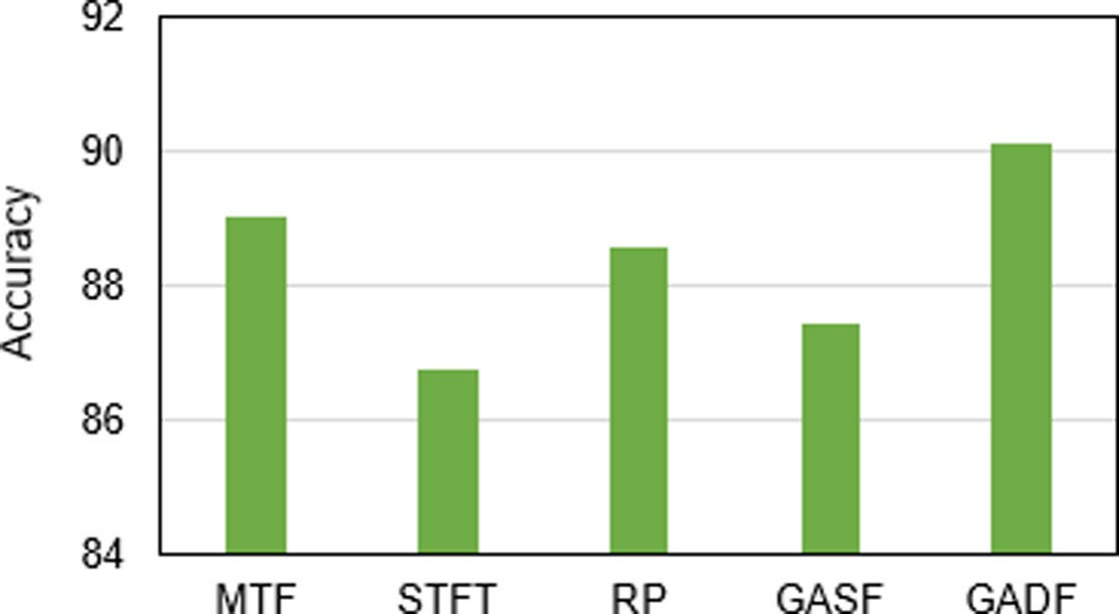
Performance comparison of different time-series-to-image representation methods.

We also conducted comparative experiments with several widely used time-series-to-image transformation methods, including Markov Transition Field (MTF), Short-Time Fourier Transform (STFT)-based spectrograms, Recurrence Plot (RP), and Gramian Angular Summation Field (GASF). All methods were evaluated under the same network architecture and training settings to ensure a fair comparison. As shown in Figure 12, the proposed GADF representation achieves the best performance among all compared methods. In particular, GADF outperforms MTF, STFT, RP, and GASF by a clear margin, demonstrating its stronger capability in preserving temporal dependencies and capturing dynamic variations in inertial signals. Overall, these results confirm that the proposed GADF-based representation is more suitable for transforming IMU signals into discriminative image-like features for cow behavior recognition tasks.

### 3.6 Comparison with other methods

To evaluate the effectiveness of the proposed method, it is compared with several representative cow behavior recognition approaches, including traditional machine learning and deep learning–based models. All methods are evaluated on the same dataset under identical experimental conditions, and the results are summarized in Table 4. For fairness, all comparison methods are implemented using their best-performing configurations, ensuring that each method can achieve its optimal performance under a consistent evaluation protocol.

**Table 4:** Performance comparison with other methods.

Reference	Method	Feeding (%)	Rumination (%)	Moving (%)	Overall (%)
Martiskainen P (29)	SVM	77.36	79.82	85.14	81.02
Vázquez Diosdado J (27)	Decision tree	80.41	82.95	86.88	83.96
Liu M (30)	FCN	83.72	85.91	89.34	86.78
Peng Y (31)	LSTM-RNN	85.74	87.63	90.18	88.19
Russel N (24)	Improved CNN	83.15	84.96	88.92	86.21
Luptáková I (32)	Transformer	86.92	87.41	90.67	88.35
Lu Z (33)	GaitFormer	87.05	87.73	90.82	88.71
Miao M (34)	Attention-Based CNN-BiGRU-Transformer	87.98	86.64	87.25	87.23
This work	GADF+ GFENet	90.32	88.29	90.03	90.12

As shown in Table 4, traditional methods such as SVM (5) and decision tree (24) achieve relatively low accuracies due to their reliance on handcrafted features and limited capability in modeling temporal dependencies. Deep learning methods, including FCN (25), LSTM-RNN (18), and improved CNN (8), show improved performance by learning hierarchical and sequential representations from inertial signals, with overall accuracies ranging from 86.21 to 88.19%. Recent Transformer-based and hybrid models (20, 26, 27) further improve performance by capturing long-range dependencies, achieving accuracies up to 88.71%. However, they still show limited ability in distinguishing fine-grained behaviors such as feeding and rumination. In contrast, as shown in Table 4, the proposed GADF + GFENet achieves the best performance with an overall accuracy of 90.12%, outperforming all comparison methods.

The performance improvement can be attributed to the use of GADF, which converts time-series signals into structured spatial representations and better captures long-range temporal dependencies than sequential models such as LSTM-RNN. In addition, GFENet enables effective fusion of global and local features through multi-scale and cross-attention mechanisms, improving the discrimination of subtle behavioral differences such as feeding and rumination.

### 3.7 Evaluation on other datasets

To further evaluate the generalization ability of the proposed method, experiments were conducted on the Frontiers IoT Collar IMU Dataset (28), a publicly available dataset for cow behavior recognition based on wearable sensors. This dataset comprises nine categories of daily behaviors recorded from ten dairy cows using neck-mounted wearable sensors.

As shown in Table 5, traditional methods such as SVM (6) already achieve relatively high accuracy (96.29%), while deep learning methods, including FCN (25), LSTM-RNN (18), and improved CNN (8), further improve performance to above 97%. Transformer-based and hybrid models (20, 26, 27) also demonstrate competitive results, with the best existing method [GaitFormer (27)] reaching 98.03%. In comparison, the proposed GADF + GFENet achieves the highest accuracy of 98.76%, indicating its strong generalization capability and effectiveness on different IMU-based datasets.

**Table 5:** Performance comparison on the Frontiers IoT Collar IMU Dataset.

Reference	Method	Accuracy
Morales-Vargas D (28)	SVM	96.29
Liu M (30)	FCN	97.12
Peng Y (31)	LSTM-RNN	97.85
Russel N (24)	Improved CNN	97.34
Luptáková I (32)	Transformer	97.83
Lu Z (33)	GaitFormer	98.03
Miao M (34)	Attention-Based CNN-BiGRU-Transformer	97.41
This work	GADF+ GFENet	98.76

## 4 Discussion

In this study, we proposed a cow behavior recognition framework based on GADF-based time-series-to-image encoding and a global–local feature enhancement network (GFENet). Experimental results demonstrate that the proposed method achieves an overall recognition accuracy of 90.12% on the test set, outperforming comparison methods such as SVM, LSTM-RNN, and Transformer-based models, thereby validating the effectiveness of the proposed approach.

The GADF time-series image encoding strategy is one of the core innovations of this study. After transforming one-dimensional acceleration signals into two-dimensional pseudo-color images, different behaviors exhibit visually distinguishable structural patterns in the image domain: feeding presents dense and repetitive textures, rumination displays regular and structured band-like patterns, and moving shows irregular and fragmented textures. This transformation reformulates the behavior recognition problem as an image classification task, enabling CNN models to fully leverage their spatial feature extraction capabilities. The comparative experiment in Figure 11 further supports this finding: all CNN models achieved higher accuracy when using GADF-based image inputs compared to raw time-series data, indicating that the proposed encoding strategy effectively preserves and enhances discriminative information within temporal signals.

The GFENet architecture is another key factor contributing to the improved recognition performance. This network captures macro-level trends and long-range dependencies of behavioral signals through a global feature extraction branch, focuses on fine-grained motion pattern features through a local branch, and introduces a Multi-Head Cross-Attention Coupling (MCC) module to achieve adaptive interaction and fusion between global and local features. Ablation study results show that the full model (B1) achieves the best performance (89.65%), while retaining only the global branch (B2) or only the local branch (B3) leads to significant performance degradation, confirming the complementary nature of the two types of features. Replacing the MCC module with simple feature concatenation (B4) also results in performance loss, demonstrating that the cross-attention-driven interaction mechanism is essential for effective feature fusion. This design effectively addresses the limitation of traditional methods, which struggle to simultaneously model global temporal structure and local detailed features.

Despite the promising results, several limitations remain. First, there is a misclassification rate of approximately 5–6% between feeding and rumination behaviors, primarily due to the high similarity in jaw movement patterns. Future work may incorporate acoustic or electromyographic signals for multimodal recognition. Second, the generalization capability of the model across different farms and breeds requires further validation, particularly under larger and more diverse populations, as the current study is based on a relatively limited number of animals. In addition, we will focus on lightweight model deployment and larger-scale data collection for further validation.

## 5 Conclusion

This study presents an effective cow behavior recognition framework based on time-series image encoding and a global–local feature enhancement network. By converting inertial signals into GADF images and leveraging complementary global and local feature representations, the proposed method improves the discriminative modeling of feeding, rumination, and moving behaviors. Experimental results on a real-world dairy farm dataset demonstrate that the proposed approach achieves an overall recognition accuracy of 90.12%, outperforming several representative traditional and deep learning–based methods. The comparative and ablation studies further confirm the effectiveness of the adopted signal representation and feature modeling strategies.

The proposed framework is not limited to laboratory-level experiments, but has potential to be integrated into real-time livestock monitoring systems for intelligent farm management and animal welfare assessment. Based on a wearable IMU sensor integrated into a nose-ring device, the system enables continuous and non-invasive acquisition of cattle motion data. The collected data are processed to automatically recognize key behaviors, including feeding, rumination, and movement. In practical farm applications, the proposed approach can serve as an intelligent behavior monitoring tool for precision livestock farming. Specifically, the behavior recognition results can be used to monitor daily activity patterns such as feeding duration and rumination cycles, which are important indicators of animal health status. Abnormal behavioral patterns may further provide early warnings of potential health issues, enabling timely intervention and improved herd management efficiency.

## Data Availability

The original contributions presented in the study are included in the article/supplementary material, further inquiries can be directed to the corresponding author/s.
